# Methyl (*E*)-2-[(2-nitro­phen­oxy)meth­yl]-3-phenyl­acrylate

**DOI:** 10.1107/S1600536812021009

**Published:** 2012-05-16

**Authors:** T. Anuradha, A. Devaraj, P. R. Seshadri, M. Bakthadoss

**Affiliations:** a Post Graduate & Research Department of Physics, Agurchand Manmull Jain College, Chennai 600 114, India; b Department of Organic Chemistry, University of Madras, Guindy Campus, Chennai 600 025, India

## Abstract

The title compound, C_17_H_15_NO_5_, adopts an *E* conformation with respect to the C=C double bond of the phenyl­acrylate unit. The phenyl ring and methyl acrylate group of the phenyl­acrylate unit are disordered over two sets of sites with site-occupancy ratios of 0.705 (5):0.295 (5) and 0.683 (3):0.317 (3), respectively. The mean plane through the benzene ring of the phenyl acrylate makes dihedral angles of 88.4 (8) (major component) and 86.7 (8)° (minor component) with the nitro­phen­oxy ring; the dihedral angle between the two components is 3.64 (6)°. Intra­molecular C—H⋯O interactions stabilise the molecular structure. In the crystal, C—H⋯O inter­actions result in a chain of mol­ecules running along the *b* axis.

## Related literature

For the industrial importance of methyl *trans*-cinnamates, see: Bhatia *et al.* (2007 ▶); Huang *et al.* (2009 ▶); Sharma (2011 ▶). For related structures, see: Anuradha *et al.* (2011 ▶); Wang *et al.* (2011 ▶). For graph-set notation, see: Bernstein *et al.* (1995 ▶). For background to the synthesis, see: Bakthadoss *et al.* (2009 ▶).
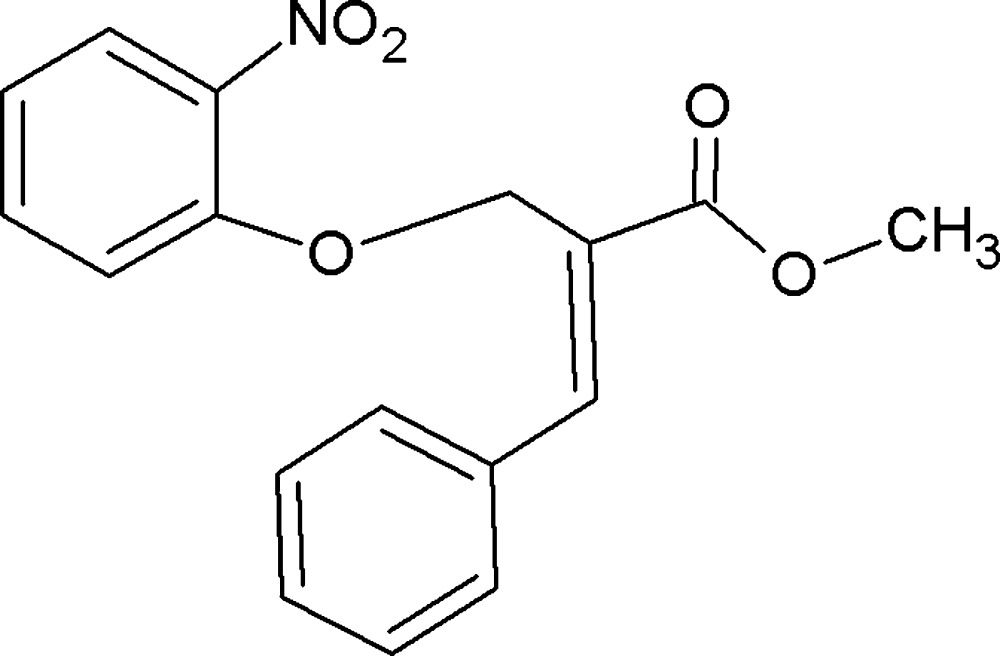


## Experimental

### 

#### Crystal data

C_17_H_15_NO_5_
*M* *_r_* = 313.30
Monoclinic, 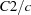

*a* = 24.0511 (10) Å
*b* = 7.8521 (3) Å
*c* = 19.7403 (9) Å
β = 121.661 (3)°
*V* = 3173.1 (2) Å^3^
*Z* = 8
Mo *K*α radiation
μ = 0.10 mm^−1^
*T* = 293 K
0.30 × 0.20 × 0.20 mm

#### Data collection

Bruker SMART APEXII area-detector diffractometer
Absorption correction: multi-scan (*SADABS*; Bruker, 2008 ▶) *T*_min_ = 0.971, *T*_max_ = 0.981
32853 measured reflections
3695 independent reflections
2356 reflections with *I* > 2σ(*I*)
*R*_int_ = 0.031

#### Refinement

*R*[*F* ^2^ > 2σ(*F* ^2^)] = 0.052
*wR*(*F* ^2^) = 0.145
*S* = 1.12
3695 reflections
212 parameters
H-atom parameters constrained
Δρ_max_ = 0.18 e Å^−3^
Δρ_min_ = −0.23 e Å^−3^

### 

Data collection: *APEX2* (Bruker, 2008 ▶); cell refinement: *SAINT* (Bruker, 2008 ▶); data reduction: *SAINT*; program(s) used to solve structure: *SHELXS97* (Sheldrick, 2008 ▶); program(s) used to refine structure: *SHELXL97* (Sheldrick, 2008 ▶); molecular graphics: *ORTEP-3* (Farrugia, 1997 ▶); software used to prepare material for publication: *PLATON* (Spek, 2009 ▶) and *publCIF* (Westrip, 2010 ▶).

## Supplementary Material

Crystal structure: contains datablock(s) I, global. DOI: 10.1107/S1600536812021009/pv2539sup1.cif

Structure factors: contains datablock(s) I. DOI: 10.1107/S1600536812021009/pv2539Isup2.hkl

Supplementary material file. DOI: 10.1107/S1600536812021009/pv2539Isup3.cml

Additional supplementary materials:  crystallographic information; 3D view; checkCIF report

## Figures and Tables

**Table 1:** Hydrogen-bond geometry (Å, °)

*D*—H⋯*A*	*D*—H	H⋯*A*	*D*⋯*A*	*D*—H⋯*A*
C9—H9⋯O3	0.93	2.26	2.683 (5)	107
C11—H11⋯O5	0.93	2.51	3.2734 (17)	140
C2—H2⋯O3^i^	0.93	2.56	3.140 (4)	121
C3—H3⋯O3^i^	0.93	2.51	3.114 (5)	123
C4—H4⋯O2^ii^	0.93	2.56	3.255 (2)	132

Symmetry codes: (i) 
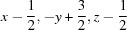
; (ii) 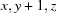.

**Fig. 1.**
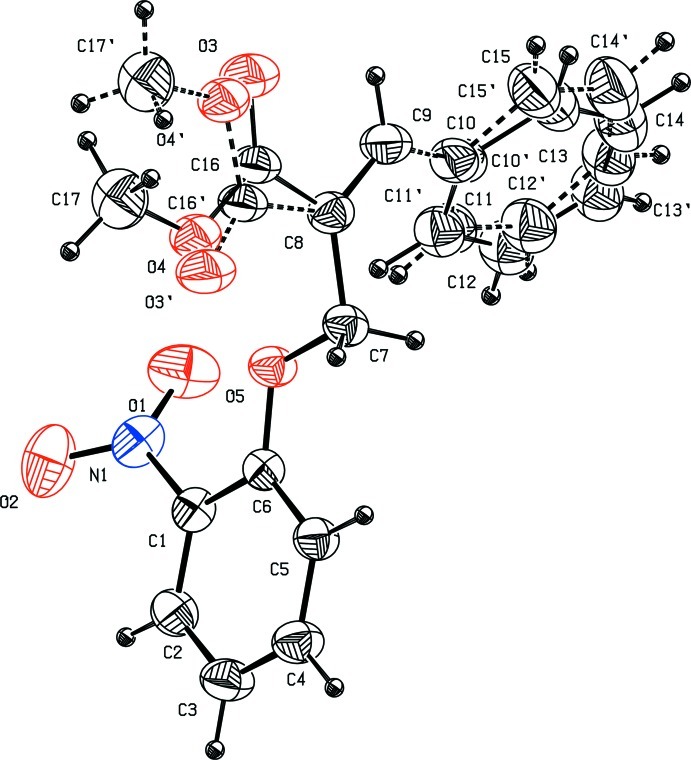
Molecular structure of the title compound, showing the atom - numbering scheme with 30% probability displacement ellipsoids. H atoms are shown as spheres of arbitrary radius. The minor fractions of the disordered benzene ring and methylacrylate have been represented by broken bonds.

**Fig. 2.**
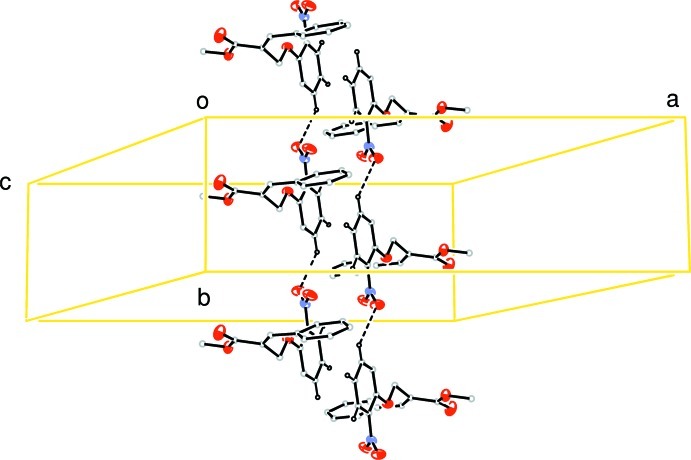
A view of the C—-H···O hydrogen bonds (dotted lines) in the crystal structure of the title compound.

## supplementary crystallographic information

### Comment

Methyl *trans*-cinnamate can inhibit both monophenolase activity and diphenolase activity of tyrosinase and thus it can be a potential compound used in antibrowning food additive (Huang *et al.*, 2009). It is a fragrance ingredient used in many fragrances and decorative cosmetics (Bhatia *et al.*, 2007; Sharma, 2011). In view of this industrial importance, we have prepared the title compound which is a nitrophenoxymethyl derivative of methyl *trans*-cinnamate and determined its crystal structure which is presented in this paper.

The title molecule adopts an E configuration with respect to the C8═C9 double bond (Fig. 1). The benzene ring (C10–C15) and methyl acrylate (C16/C17/O3/O4) group of the phenylacrylate unit are disordered over two orientations with site-occupancy ratios of 0.705 (5):0.295 (5) and 0.683 (3):0.317 (3) representing major and minor components, repectively. The mean plane through the benzene ring of the phenyl acrylate makes dihedral angles of 88.4 (8) (major component) and 86.7 (8)° (minor component) with the nitrophenoxy (C1–C6/N1/O1/O2) ring; the dihedral angle between the two components is 3.6 (6)°.

The major and minor components of the methylacrylate (C8/C16/C17/O3/O4) are essentially planar with maximum deviations for atoms O4 and O4', -0.015 (1) and 0.015 (1) Å, respectively. The central unit (C6–C8/O5) is almost equatorial to the major component of methylphenylacrylate group (C8–C17/O1/O2) whereas axial to the nitrobenzene (C1–C6/N1), making dihedral angles of 88.4 (1) and 8.1 (1)°, respectively.

The crystal structure is stabilized by intramolecular bifurcated C—H···O hydrogen bonds involving two hydrogen atoms (H2/H3) of the benzene ring (C1—C6) and O3 of the acrylate resulting in an *R^2^_2_*(5) ring motif (Bernstein *et al.*, 1995) and C4—H4···O2 interactions resulting in a chain of molecules running along the *b*-axis (Table 1 and Fig. 2).

The crystal structures of a few related compounds have been reported recently (Anuradha *et al.*, 2011); Wang *et al.*, 2011).

### Experimental

To a stirred solution of 2-nitrophenol (0.14 g, 1 mmol) in acetonitrile (7 ml), potassium carbonate (0.35 g, 2.5 mmol) was added and stirred well for five minutes. To this solution, (*Z*)- methyl 2-(bromomethyl)-3-phenylacrylate (0.26 g, 1 mmol) in acetonitrile (0.5 ml) was added and allowed to stir well for 6 h. After the completion of the reaction, the reaction mixture was poured into water and extracted using ethyl acetate. The organic layer thus obtained was concentrated under reduced pressure and the residual mass thus obtained was purified by column chromatography on silica gel (Acme 100–200) using EtOAc-hexanes (1:9) to afford the title compound in 90% yield. The crystals suitable for X-ray crystallographic analysis were grown from a solution of ethylacetate by slow evaporation at room temperature.

### Refinement

The benzene ring(C10 - C15) and methyl acrylate(C16/C17/O3/O4) group of the phenylacrylate unit are disordered over two orientations with site-occupancy ratio of 0.705 (5):0.295 (5) and 0.683 (3):0.317 (3) representing major and minor components repectively. The command EADP was used in SHELXL-97 (Sheldrick, 2008) to constrain the *U*_eq_ of the disordered atoms. The hydrogen atoms were placed in calculated positions with C—H = 0.93, 0.96 and 0.97 Å, for acryl, methyl and methylene H-atoms, respectively, and refined in the riding mode; the *U*_iso_(H) were allowed at 1.5*U*_eq_(C methyl) or 1.2*U*_eq_(C non-methyl).

### Crystal data

**Table:**

C_17_H_15_NO_5_	*F*(000) = 1312
*M**_r_* = 313.30	*D*_x_ = 1.312 Mg m^−^^3^
Monoclinic, *C*2/*c*	Mo *K*α radiation, λ = 0.71073 Å
Hall symbol: -C 2yc	Cell parameters from 3695 reflections
*a* = 24.0511 (10) Å	θ = 2.2–27.7°
*b* = 7.8521 (3) Å	µ = 0.10 mm^−^^1^
*c* = 19.7403 (9) Å	*T* = 293 K
β = 121.661 (3)°	Block, colourless
*V* = 3173.1 (2) Å^3^	0.30 × 0.20 × 0.20 mm
*Z* = 8	

### Data collection

**Table:**

Bruker SMART APEXII area-detector diffractometer	3695 independent reflections
Radiation source: fine-focus sealed tube	2356 reflections with *I* > 2σ(*I*)
Graphite monochromator	*R*_int_ = 0.031
ω and φ scans	θ_max_ = 27.7°, θ_min_ = 2.2°
Absorption correction: multi-scan (*SADABS*; Bruker, 2008)	*h* = −31→31
*T*_min_ = 0.971, *T*_max_ = 0.981	*k* = −10→10
32853 measured reflections	*l* = −25→25

### Refinement

**Table:**

Refinement on *F*^2^	Primary atom site location: structure-invariant direct methods
Least-squares matrix: full	Secondary atom site location: difference Fourier map
*R*[*F*^2^ > 2σ(*F*^2^)] = 0.052	Hydrogen site location: inferred from neighbouring sites
*wR*(*F*^2^) = 0.145	H-atom parameters constrained
*S* = 1.12	*w* = 1/[σ^2^(*F*_o_^2^) + (0.0548*P*)^2^ + 1.2534*P*] where *P* = (*F*_o_^2^ + 2*F*_c_^2^)/3
3695 reflections	(Δ/σ)_max_ = 0.008
212 parameters	Δρ_max_ = 0.18 e Å^−^^3^
0 restraints	Δρ_min_ = −0.23 e Å^−^^3^

### Special details

**Table:**

Geometry. All e.s.d.'s (except the e.s.d. in the dihedral angle between two l.s. planes) are estimated using the full covariance matrix. The cell e.s.d.'s are taken into account individually in the estimation of e.s.d.'s in distances, angles and torsion angles; correlations between e.s.d.'s in cell parameters are only used when they are defined by crystal symmetry. An approximate (isotropic) treatment of cell e.s.d.'s is used for estimating e.s.d.'s involving l.s. planes.
Refinement. Refinement of *F*^2^ against ALL reflections. The weighted *R*-factor *wR* and goodness of fit *S* are based on *F*^2^, conventional *R*-factors *R* are based on *F*, with *F* set to zero for negative *F*^2^. The threshold expression of *F*^2^ > σ(*F*^2^) is used only for calculating *R*-factors(gt) *etc*. and is not relevant to the choice of reflections for refinement. *R*-factors based on *F*^2^ are statistically about twice as large as those based on *F*, and *R*- factors based on ALL data will be even larger.

### Fractional atomic coordinates and isotropic or equivalent isotropic displacement parameters (Å^2^)

**Table:**

	*x*	*y*	*z*	*U*_iso_*/*U*_eq_	Occ. (<1)
C1	0.04775 (8)	0.71127 (19)	−0.03978 (10)	0.0530 (4)	
C2	−0.00796 (9)	0.7393 (2)	−0.11206 (11)	0.0646 (5)	
H2	−0.0326	0.6479	−0.1435	0.078*	
C3	−0.02733 (9)	0.9032 (2)	−0.13791 (12)	0.0722 (5)	
H3	−0.0652	0.9239	−0.1868	0.087*	
C4	0.00989 (9)	1.0359 (2)	−0.09087 (11)	0.0684 (5)	
H4	−0.0035	1.1470	−0.1080	0.082*	
C5	0.06650 (8)	1.0086 (2)	−0.01904 (10)	0.0581 (4)	
H5	0.0912	1.1008	0.0115	0.070*	
C6	0.08709 (7)	0.84407 (19)	0.00821 (9)	0.0494 (4)	
C7	0.18319 (8)	0.9397 (2)	0.12608 (10)	0.0561 (4)	
H7A	0.1617	0.9959	0.1501	0.067*	
H7B	0.1897	1.0228	0.0945	0.067*	
C8	0.24735 (8)	0.8703 (2)	0.18938 (11)	0.0598 (4)	
C9	0.26255 (9)	0.8346 (2)	0.26281 (11)	0.0698 (5)	
H9	0.3061	0.8069	0.2985	0.084*	
O5	0.14319 (5)	0.80287 (13)	0.07619 (7)	0.0586 (3)	
N1	0.06558 (8)	0.53526 (19)	−0.01355 (12)	0.0696 (4)	
O1	0.08558 (8)	0.49972 (19)	0.05526 (11)	0.1018 (6)	
O2	0.05728 (10)	0.43151 (19)	−0.06366 (12)	0.1139 (6)	
O3	0.35656 (19)	0.8059 (6)	0.2290 (2)	0.1002 (11)	0.683 (3)
O4	0.28384 (12)	0.8651 (4)	0.1015 (2)	0.0720 (7)	0.683 (3)
C17	0.33209 (17)	0.8375 (5)	0.0840 (2)	0.0942 (9)	0.683 (3)
H17A	0.3619	0.9319	0.1028	0.141*	0.683 (3)
H17B	0.3123	0.8267	0.0274	0.141*	0.683 (3)
H17C	0.3554	0.7348	0.1095	0.141*	0.683 (3)
C16	0.30259 (19)	0.8415 (5)	0.1786 (2)	0.0616 (8)	0.683 (3)
O3'	0.2676 (4)	0.9073 (12)	0.0829 (6)	0.1002 (11)	0.317 (3)
O4'	0.3504 (4)	0.8074 (11)	0.2006 (4)	0.0720 (7)	0.317 (3)
C17'	0.3931 (4)	0.8028 (11)	0.1689 (5)	0.0942 (9)	0.317 (3)
H17D	0.3785	0.7156	0.1291	0.141*	0.317 (3)
H17E	0.4369	0.7786	0.2112	0.141*	0.317 (3)
H17F	0.3919	0.9111	0.1457	0.141*	0.317 (3)
C16'	0.2889 (5)	0.8675 (13)	0.1467 (6)	0.0616 (8)	0.317 (3)
C10	0.21965 (15)	0.8329 (3)	0.29588 (16)	0.0693 (5)	0.705 (5)
C11	0.15778 (8)	0.76028 (14)	0.24978 (8)	0.0740 (6)	0.705 (5)
H11	0.1441	0.7189	0.1992	0.089*	0.705 (5)
C12	0.11639 (8)	0.74947 (14)	0.27897 (8)	0.0868 (8)	0.705 (5)
H12	0.0750	0.7020	0.2478	0.104*	0.705 (5)
C13	0.13684 (8)	0.80956 (14)	0.35480 (8)	0.0957 (11)	0.705 (5)
H13	0.1091	0.8023	0.3743	0.115*	0.705 (5)
C14	0.19868 (8)	0.88046 (14)	0.40143 (8)	0.1047 (12)	0.705 (5)
H14	0.2124	0.9207	0.4522	0.126*	0.705 (5)
C15	0.24007 (8)	0.89128 (14)	0.37224 (8)	0.0906 (8)	0.705 (5)
H15	0.2816	0.9377	0.4038	0.109*	0.705 (5)
C10'	0.22131 (8)	0.82692 (14)	0.29177 (8)	0.0693 (5)	0.295 (5)
C11'	0.15763 (8)	0.76783 (14)	0.25774 (8)	0.0740 (6)	0.295 (5)
H11'	0.1350	0.7290	0.2054	0.089*	0.295 (5)
C12'	0.12776 (8)	0.76678 (14)	0.30186 (8)	0.0868 (8)	0.295 (5)
H12'	0.0851	0.7272	0.2791	0.104*	0.295 (5)
C13'	0.16156 (8)	0.82481 (14)	0.38001 (8)	0.0957 (11)	0.295 (5)
H13'	0.1416	0.8241	0.4095	0.115*	0.295 (5)
C14'	0.22524 (8)	0.88390 (14)	0.41404 (8)	0.1047 (12)	0.295 (5)
H14'	0.2479	0.9227	0.4663	0.126*	0.295 (5)
C15'	0.25511 (8)	0.88495 (14)	0.36992 (8)	0.0906 (8)	0.295 (5)
H15'	0.2977	0.9245	0.3927	0.109*	0.295 (5)

### Atomic displacement parameters (Å^2^)

**Table:**

	*U*^11^	*U*^22^	*U*^33^	*U*^12^	*U*^13^	*U*^23^
C1	0.0514 (9)	0.0474 (8)	0.0629 (10)	−0.0054 (7)	0.0317 (8)	−0.0016 (7)
C2	0.0574 (10)	0.0612 (10)	0.0651 (11)	−0.0131 (8)	0.0252 (9)	−0.0118 (8)
C3	0.0590 (11)	0.0702 (12)	0.0622 (11)	−0.0030 (9)	0.0144 (9)	0.0014 (9)
C4	0.0630 (11)	0.0555 (9)	0.0670 (12)	0.0017 (8)	0.0206 (10)	0.0051 (8)
C5	0.0567 (10)	0.0485 (8)	0.0574 (10)	−0.0040 (7)	0.0219 (8)	−0.0024 (7)
C6	0.0476 (9)	0.0510 (8)	0.0501 (9)	−0.0043 (6)	0.0260 (8)	−0.0011 (7)
C7	0.0545 (9)	0.0500 (8)	0.0536 (9)	−0.0059 (7)	0.0213 (8)	−0.0018 (7)
C8	0.0514 (9)	0.0539 (9)	0.0609 (11)	−0.0018 (7)	0.0204 (8)	−0.0026 (8)
C9	0.0615 (11)	0.0590 (10)	0.0628 (12)	0.0052 (8)	0.0146 (9)	0.0041 (8)
O5	0.0540 (7)	0.0487 (6)	0.0561 (7)	−0.0037 (5)	0.0172 (6)	0.0015 (5)
N1	0.0605 (9)	0.0495 (8)	0.0984 (13)	−0.0112 (7)	0.0414 (9)	−0.0019 (8)
O1	0.1010 (12)	0.0756 (10)	0.1010 (12)	−0.0169 (8)	0.0338 (10)	0.0265 (9)
O2	0.1455 (16)	0.0531 (8)	0.1600 (17)	−0.0084 (9)	0.0919 (14)	−0.0197 (10)
O3	0.0626 (15)	0.140 (2)	0.078 (2)	0.0263 (14)	0.0233 (18)	0.008 (2)
O4	0.0476 (14)	0.0938 (16)	0.069 (2)	0.0054 (10)	0.0265 (15)	0.0008 (13)
C17	0.086 (2)	0.120 (3)	0.099 (2)	−0.0060 (19)	0.0632 (18)	−0.003 (2)
C16	0.049 (2)	0.0636 (17)	0.051 (3)	0.0007 (14)	0.012 (2)	0.0024 (17)
O3'	0.0626 (15)	0.140 (2)	0.078 (2)	0.0263 (14)	0.0233 (18)	0.008 (2)
O4'	0.0476 (14)	0.0938 (16)	0.069 (2)	0.0054 (10)	0.0265 (15)	0.0008 (13)
C17'	0.086 (2)	0.120 (3)	0.099 (2)	−0.0060 (19)	0.0632 (18)	−0.003 (2)
C16'	0.049 (2)	0.0636 (17)	0.051 (3)	0.0007 (14)	0.012 (2)	0.0024 (17)
C10	0.0848 (14)	0.0554 (10)	0.0554 (11)	0.0092 (9)	0.0284 (10)	0.0094 (8)
C11	0.0916 (15)	0.0683 (12)	0.0651 (12)	0.0022 (10)	0.0432 (12)	0.0089 (9)
C12	0.106 (2)	0.0881 (16)	0.0731 (18)	0.0032 (14)	0.0516 (17)	0.0131 (14)
C13	0.127 (3)	0.0971 (19)	0.079 (2)	0.009 (2)	0.065 (2)	0.0098 (17)
C14	0.136 (4)	0.106 (2)	0.084 (2)	−0.004 (2)	0.066 (3)	−0.0063 (16)
C15	0.109 (2)	0.0903 (17)	0.0643 (13)	−0.0018 (14)	0.0402 (15)	−0.0044 (12)
C10'	0.0848 (14)	0.0554 (10)	0.0554 (11)	0.0092 (9)	0.0284 (10)	0.0094 (8)
C11'	0.0916 (15)	0.0683 (12)	0.0651 (12)	0.0022 (10)	0.0432 (12)	0.0089 (9)
C12'	0.106 (2)	0.0881 (16)	0.0731 (18)	0.0032 (14)	0.0516 (17)	0.0131 (14)
C13'	0.127 (3)	0.0971 (19)	0.079 (2)	0.009 (2)	0.065 (2)	0.0098 (17)
C14'	0.136 (4)	0.106 (2)	0.084 (2)	−0.004 (2)	0.066 (3)	−0.0063 (16)
C15'	0.109 (2)	0.0903 (17)	0.0643 (13)	−0.0018 (14)	0.0402 (15)	−0.0044 (12)

### Geometric parameters (Å, º)

**Table:**

C1—C2	1.369 (2)	C17—H17C	0.9600
C1—C6	1.392 (2)	O3'—C16'	1.126 (14)
C1—N1	1.459 (2)	O4'—C16'	1.375 (14)
C2—C3	1.372 (3)	O4'—C17'	1.456 (9)
C2—H2	0.9300	C17'—H17D	0.9600
C3—C4	1.370 (2)	C17'—H17E	0.9600
C3—H3	0.9300	C17'—H17F	0.9600
C4—C5	1.372 (2)	C10—C15	1.395 (3)
C4—H4	0.9300	C10—C11	1.395 (4)
C5—C6	1.387 (2)	C11—C12	1.3900
C5—H5	0.9300	C11—H11	0.9300
C6—O5	1.3510 (18)	C12—C13	1.3900
C7—O5	1.4334 (18)	C12—H12	0.9300
C7—C8	1.489 (2)	C13—C14	1.3900
C7—H7A	0.9700	C13—H13	0.9300
C7—H7B	0.9700	C14—C15	1.3900
C8—C9	1.325 (3)	C14—H14	0.9300
C8—C16	1.469 (5)	C15—H15	0.9300
C8—C16'	1.610 (13)	C10'—C11'	1.3900
C9—C10'	1.382 (3)	C10'—C15'	1.3900
C9—C10	1.483 (4)	C11'—C12'	1.3900
C9—H9	0.9300	C11'—H11'	0.9300
N1—O1	1.212 (2)	C12'—C13'	1.3900
N1—O2	1.214 (2)	C12'—H12'	0.9300
O3—C16	1.182 (5)	C13'—C14'	1.3900
O4—C16	1.353 (4)	C13'—H13'	0.9300
O4—C17	1.392 (4)	C14'—C15'	1.3900
C17—H17A	0.9600	C14'—H14'	0.9300
C17—H17B	0.9600	C15'—H15'	0.9300
			
C2—C1—C6	122.17 (15)	O4'—C17'—H17E	109.5
C2—C1—N1	117.84 (15)	H17D—C17'—H17E	109.5
C6—C1—N1	119.98 (15)	O4'—C17'—H17F	109.5
C1—C2—C3	119.61 (16)	H17D—C17'—H17F	109.5
C1—C2—H2	120.2	H17E—C17'—H17F	109.5
C3—C2—H2	120.2	O3'—C16'—O4'	129.3 (12)
C4—C3—C2	119.18 (17)	O3'—C16'—C8	122.6 (9)
C4—C3—H3	120.4	O4'—C16'—C8	108.1 (7)
C2—C3—H3	120.4	C15—C10—C11	119.3 (3)
C3—C4—C5	121.49 (17)	C15—C10—C9	122.8 (2)
C3—C4—H4	119.3	C11—C10—C9	117.9 (2)
C5—C4—H4	119.3	C12—C11—C10	120.36 (15)
C4—C5—C6	120.32 (15)	C12—C11—H11	119.8
C4—C5—H5	119.8	C10—C11—H11	119.8
C6—C5—H5	119.8	C11—C12—C13	120.0
O5—C6—C5	125.12 (14)	C11—C12—H12	120.0
O5—C6—C1	117.65 (14)	C13—C12—H12	120.0
C5—C6—C1	117.19 (14)	C12—C13—C14	120.0
O5—C7—C8	109.18 (13)	C12—C13—H13	120.0
O5—C7—H7A	109.8	C14—C13—H13	120.0
C8—C7—H7A	109.8	C13—C14—C15	120.0
O5—C7—H7B	109.8	C13—C14—H14	120.0
C8—C7—H7B	109.8	C15—C14—H14	120.0
H7A—C7—H7B	108.3	C14—C15—C10	120.35 (15)
C9—C8—C16	112.0 (2)	C14—C15—H15	119.8
C9—C8—C7	124.38 (17)	C10—C15—H15	119.8
C16—C8—C7	123.5 (2)	C9—C10'—C11'	131.51 (10)
C9—C8—C16'	132.8 (4)	C9—C10'—C15'	108.43 (10)
C7—C8—C16'	102.7 (4)	C11'—C10'—C15'	120.0
C8—C9—C10'	128.02 (17)	C12'—C11'—C10'	120.0
C8—C9—C10	128.86 (19)	C12'—C11'—H11'	120.0
C8—C9—H9	115.6	C10'—C11'—H11'	120.0
C10'—C9—H9	116.4	C11'—C12'—C13'	120.0
C10—C9—H9	115.6	C11'—C12'—H12'	120.0
C6—O5—C7	117.59 (12)	C13'—C12'—H12'	120.0
O1—N1—O2	123.87 (18)	C14'—C13'—C12'	120.0
O1—N1—C1	119.05 (16)	C14'—C13'—H13'	120.0
O2—N1—C1	117.03 (18)	C12'—C13'—H13'	120.0
C16—O4—C17	115.4 (3)	C13'—C14'—C15'	120.0
O3—C16—O4	123.3 (4)	C13'—C14'—H14'	120.0
O3—C16—C8	126.3 (3)	C15'—C14'—H14'	120.0
O4—C16—C8	110.4 (3)	C10'—C15'—C14'	120.0
C16'—O4'—C17'	113.0 (8)	C10'—C15'—H15'	120.0
O4'—C17'—H17D	109.5	C14'—C15'—H15'	120.0
			
C6—C1—C2—C3	−1.9 (3)	C16'—C8—C16—O4	−9.8 (12)
N1—C1—C2—C3	178.29 (17)	C17'—O4'—C16'—O3'	2.5 (17)
C1—C2—C3—C4	0.4 (3)	C17'—O4'—C16'—C8	−178.7 (6)
C2—C3—C4—C5	0.9 (3)	C9—C8—C16'—O3'	−177.7 (8)
C3—C4—C5—C6	−0.8 (3)	C16—C8—C16'—O3'	176 (2)
C4—C5—C6—O5	177.10 (16)	C7—C8—C16'—O3'	−2.1 (12)
C4—C5—C6—C1	−0.6 (3)	C9—C8—C16'—O4'	3.4 (11)
C2—C1—C6—O5	−175.93 (15)	C16—C8—C16'—O4'	−2.5 (9)
N1—C1—C6—O5	3.9 (2)	C7—C8—C16'—O4'	179.0 (6)
C2—C1—C6—C5	2.0 (2)	C8—C9—C10—C15	−141.3 (2)
N1—C1—C6—C5	−178.22 (15)	C10'—C9—C10—C15	148 (3)
O5—C7—C8—C9	−97.96 (19)	C8—C9—C10—C11	42.5 (3)
O5—C7—C8—C16	85.3 (3)	C10'—C9—C10—C11	−28 (3)
O5—C7—C8—C16'	85.9 (4)	C15—C10—C11—C12	1.3 (3)
C16—C8—C9—C10'	−171.5 (2)	C9—C10—C11—C12	177.64 (13)
C7—C8—C9—C10'	11.4 (3)	C10—C11—C12—C13	−0.67 (14)
C16'—C8—C9—C10'	−173.8 (5)	C11—C12—C13—C14	0.0
C16—C8—C9—C10	−174.4 (2)	C12—C13—C14—C15	0.0
C7—C8—C9—C10	8.5 (3)	C13—C14—C15—C10	0.66 (14)
C16'—C8—C9—C10	−176.7 (5)	C11—C10—C15—C14	−1.3 (3)
C5—C6—O5—C7	3.1 (2)	C9—C10—C15—C14	−177.45 (14)
C1—C6—O5—C7	−179.15 (14)	C8—C9—C10'—C11'	37.6 (2)
C8—C7—O5—C6	−169.94 (14)	C10—C9—C10'—C11'	149 (3)
C2—C1—N1—O1	−137.99 (18)	C8—C9—C10'—C15'	−145.33 (17)
C6—C1—N1—O1	42.2 (2)	C10—C9—C10'—C15'	−34 (3)
C2—C1—N1—O2	39.7 (2)	C9—C10'—C11'—C12'	176.81 (12)
C6—C1—N1—O2	−140.11 (18)	C15'—C10'—C11'—C12'	0.0
C17—O4—C16—O3	2.7 (6)	C10'—C11'—C12'—C13'	0.0
C17—O4—C16—C8	−178.7 (3)	C11'—C12'—C13'—C14'	0.0
C9—C8—C16—O3	−6.5 (5)	C12'—C13'—C14'—C15'	0.0
C7—C8—C16—O3	170.6 (4)	C9—C10'—C15'—C14'	−177.49 (9)
C16'—C8—C16—O3	168.8 (17)	C11'—C10'—C15'—C14'	0.0
C9—C8—C16—O4	174.8 (2)	C13'—C14'—C15'—C10'	0.0
C7—C8—C16—O4	−8.1 (4)		

### Hydrogen-bond geometry (Å, º)

**Table:**

*D*—H···*A*	*D*—H	H···*A*	*D*···*A*	*D*—H···*A*
C9—H9···O3	0.93	2.26	2.683 (5)	107
C11—H11···O5	0.93	2.51	3.2734 (17)	140
C2—H2···O3^i^	0.93	2.56	3.140 (4)	121
C3—H3···O3^i^	0.93	2.51	3.114 (5)	123
C4—H4···O2^ii^	0.93	2.56	3.255 (2)	132

Symmetry codes: (i) *x*−1/2, −*y*+3/2, *z*−1/2; (ii) *x*, *y*+1, *z*.
